# Development of a dual vaccine against East Coast fever and lumpy skin disease

**DOI:** 10.3389/fimmu.2023.1143034

**Published:** 2023-03-30

**Authors:** Leah Whittle, Ros Chapman, Nicola Douglass, Mohamed Jaffer, Emmanuel Margolin, Edward Rybicki, Anna-Lise Williamson

**Affiliations:** ^1^ Institute of Infectious Disease and Molecular Medicine, Faculty of Health Sciences, University of Cape Town, Cape Town, South Africa; ^2^ Division of Medical Virology, Department of Pathology, Faculty of Health Sciences, University of Cape Town, Cape Town, South Africa; ^3^ Electron Microscope Unit, University of Cape Town, Cape Town, South Africa; ^4^ Biopharming Research Unit, Department of Molecular and Cell Biology, Faculty of Science, University of Cape Town, Cape Town, South Africa

**Keywords:** *Theileria parva*, East Coast fever, p67, VLPs, Gag, LSDV, poxvirus, vaccine

## Abstract

East Coast fever is an acute bovine disease caused by the apicomplexan parasite *Theileria parva* and is regarded as one of the most important tick-vectored diseases in Africa. The current vaccination procedure has many drawbacks, as it involves the use of live *T. parva* sporozoites. As a novel vaccination strategy, we have constructed the recombinant lumpy skin disease virus (LSDV) named LSDV-SODis-p67HA-BLV-Gag, encoding a modified form of the *T. parva* p67 surface antigen (p67HA), as well as the bovine leukemia virus (BLV) gag gene for the formation of virus-like particles (VLPs) to potentially enhance p67 immunogenicity. In place of the native sequence, the chimeric p67HA antigen has the human tissue plasminogen activator signal sequence and the influenza hemagglutinin A2 transmembrane domain and cytoplasmic tail. p67HA was detected on the surface of infected cells, and VLPs comprising BLV Gag and p67HA were produced. We also show that higher multiple bands observed in western blot analysis are due to glycosylation of p67. The two vaccines, pMExT-p67HA (DNA) and LSDV-SODis-p67HA-BLV-Gag, were tested for immunogenicity in mice. p67-binding antibodies were produced by vaccinated animals, with higher titers detected in mice vaccinated with the recombinant LSDV. This candidate dual vaccine warrants further testing in cattle.

## 1 Introduction

Tick-borne diseases are a major challenge to cattle farmers in Africa. Of these, East Coast fever (ECF) is considered one of the most burdensome in the affected east and sub-Saharan regions (1–5). The disease is acute and characterized by a fever, respiratory distress, mucosal petechiae and recumbency. Severe cases often result in death after three weeks due to fluid build-up in the lungs and consequent respiratory failure (6, 7). The causative agent, the apicomplexan parasite *Theileria parva*, is transmitted to cattle or buffalo *via* the brown ear tick *Rhipicephalus appendiculatus* (8). Currently, cattle are immunized against ECF by the infection and treatment method (ITM) which involves the use of live *T. parva* sporozoites and the immediate administration of long-acting oxytetracycline (9). While this does provide effective protection against ECF, there are many drawbacks to this method which include 1) the production of live *T. parva* sporozoites is a lengthy process that requires cattle, rabbits and ticks, 2) liquid nitrogen is required for storage and transport of live parasites and 3) cattle immunized by this method become *T. parva* carriers and can spread the disease 4) the potential development of oxytetracycline resistance (10–13).

Other vaccine platforms have the potential to overcome the logistics of using a live unattenuated parasite as a vaccine. Lumpy skin disease virus (LSDV) would be an ideal candidate to vector *T. parva* antigens. Poxviruses are relatively stable and can be freeze-dried, they do not require animals for vaccine production and pose no risk to establishing a *T. parva* carrier state (14). In addition, their large genomes can tolerate the insertion of multiple foreign genes, and poxviruses are known to induce strong humoral and cellular immune responses (15, 16). LSDV has the added advantage of providing protection against lumpy skin disease (LSD) (17). LSD is a serious threat to the cattle industry and many African countries affected by it are also affected by ECF (8, 18). LSD is characterized by fever, ocular and nasal discharge, and painful nodular lesions (19, 20). Infertility, loss of body weight and decreased milk production also have negative economic impacts in affected regions (21). A number of live attenuated LSDV strains have been developed to protect cattle against LSD, such as the Neethling vaccine strain produced by Onderstepoort Biological Products (OBP) currently used in South Africa (22). Attenuated LSDV has been used in experimental vaccines to vector the antigens of other pathogens, not only limited to cattle, which include those of human immunodeficiency virus (HIV), rabies virus and Rift Valley fever virus (23–25). Our group has constructed an improved LSDV recombinant backbone named nLSDVSODis-UCT which is based on the Neethling vaccine strain (26). When used to vector bovine ephemeral fever virus (BEFV) antigens, vaccinated cattle produced BEFV neutralizing antibodies at titers considered protective and survived virulent LSDV challenge (17). Therefore, nLSDVSODis-UCT would be an ideal choice for a poxvirus-vectored ECF vaccine.

Many efforts have been made to develop a novel ECF vaccine using the *T. parva* major sporozoite surface protein p67 (27). The antigen induces sporozoite neutralizing antibodies in cattle, however only partial protection against *T. parva* challenge has been attained (28). Others have shown that the immunogenicity of p67 truncated to its C-terminal region (p67C) was improved when placed on the surface of baculovirus virions, hepatitis B VLPs or associated with silica vesicles, in comparison to free soluble p67C (28, 29). Therefore, we hypothesized that a less-truncated p67 displayed on a particle may provide an even further improved p67-based vaccine. We have previously attempted to improve the immunogenicity by displaying p67 with an influenza hemagglutinin A2 (HA_2_) anchor on the surface of retrovirus Gag virus-like particles (VLPs) (30). The antigen named p67HA retains all the known immunogenic regions of p67 and would potentially benefit from the immunogenic enhancement of VLP display. p67HA was characterized using DNA mammalian expression vectors and was shown to be immunogenic in mice.

DNA vaccines have previously yielded unsuccessful results in cattle (31, 32) and LSDV has been shown to be a good vaccine vector (17). We therefore constructed a recombinant LSDV, LSDV-SODis-p67HA-BLV-Gag, to express the described p67HA together with bovine leukemia virus (BLV) Gag so as to generate VLPs. Expression of the antigens were characterized by immunostaining and electron microscopy, and the immunogenicity of the recombinant LSDV was compared to the p67HA DNA vaccine in mice. We also investigated glycosylation of recombinant p67 expressed in mammalian cells using soluble purified p67.

## 2 Materials and methods

### 2.1 Cells, fertilized hens’ eggs, viruses and antibodies

Madin Darby bovine epithelial kidney cells (MDBK) (CCL-22™ ATCC^®^, USA), baby hamster kidney fibroblast 21 cells (BHK-21) (CCL-10™ ATCC, USA), HeLa cells (CCL-2™ ATCC^®^, USA) and primary fetal lamb testes (LT) cells were cultured in Dulbecco’s modified Eagle’s medium with GlutaMAX™ (DMEM) (Thermo Fisher Scientific, USA) containing 10% fetal bovine serum (FBS) (Thermo Fisher Scientific, USA, or HyClone™ Cytiva, USA for LT cells) and 1X penicillin/streptomycin (1000 U/ml each, Lonza, Belgium), at 37°C with 5% CO_2_.

Specific pathogen-free (SPF) Leghorn chicken eggs (AviFarms, RSA) were maintained in accordance with the University of Cape Town (UCT) Animal Ethics Committee (AEC) protocol AEC 018-022.

nLSDVSODis-UCT was used as the LSDV backbone and for experimental controls. The virus is based on the Neethling vaccine strain originally obtained from Onderstepoort Biological Products (OBP, RSA), which was modified to encode a synthetic, improved and stabilized superoxide dismutase gene (SODis) (26). The parent virus, LSDV(SODis)BEFV-Gb, has nLSDVSODis-UCT encoding the BEFV glycoprotein Gb antigen and eGFP, under the control of the respective vaccinia virus (VACV) mH5 and synthetic pSS poxvirus promoters, all inserted between open reading frame (ORF) 49 and 50 (17).

Rabbit polyclonal anti-p67 raised against the peptide LKKTLQPGKTSTGETC (GenScript, China) which contained the *T. parva* sporozoite neutralizing epitope recognized by the monoclonal AR22.7 (33), mouse monoclonal anti-BLV p24 (Gag) (BLV-3, VMRD, USA) and mouse monoclonal anti-X6 His (MCA1396, Bio-Rad, USA) were used as primary antibodies.

### 2.2 Design, construction and passage of the recombinant LSDV

**Figure 1:**
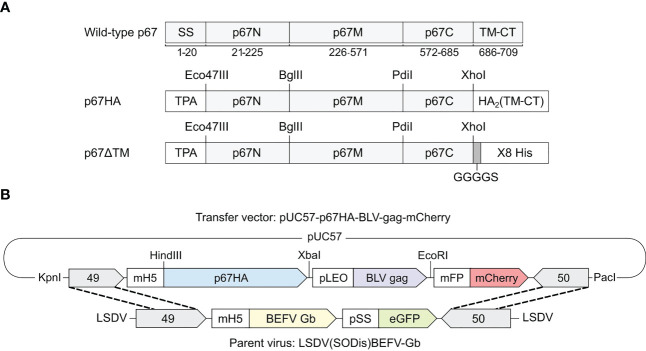
**(A)** Schematic diagrams of the recombinant antigens p67HA and p67ΔTM in comparison to wild-type p67 (Muguga, GenBank: AP018021.1) with labelled amino acid residue positions underneath. The p67N, p67M and p67C regions based on B-cell epitope distribution are annotated as previously described (34). Both p67HA and p67ΔTM had the native signal sequence (SS) replaced with that of the human tissue plasminogen activator (TPA). The native predicted transmembrane domain and cytoplasmic tail (TM-CT) were replaced with those of influenza virus A H5N1 hemagglutinin 2 (HA_2_) for p67HA or were replaced with a GGGGS linker and 8X His tag for p67ΔTM. **(B)** Schematic diagram of the transfer vector pUC57-p67HA-BLV-Gag-mCherry for homologous recombination with the parent virus LSDV(SODis)BEFV-Gb genome between ORFs 49 and 50 to generate LSDV-SODis-p67HA-BLV-Gag. Restriction enzyme sites that were used for cloning or removal of the expression cassette from pUC57-Simple are labelled.

The *T. parva* modified p67 major sporozoite surface antigens named p67HA and p67ΔTM have been described previously (30) (**Figure 1A**). The p67 amino acid regions are identical to wild-type p67 (Muguga, GenBank: AAA98601.1). p67HA is the primary immunogen in this study whereas p67ΔTM purified from HEK293T cell media was used for assays.

The transfer vector pUC57-p67HA-BLV-Gag-mCherry (**Figure 1B**) was constructed to have the genes for p67HA, BLV gag (GenBank: AP018021.1) (30) and fluorescent marker mCherry, with the respective TTTTTCT, TTTTTAT and TTTTTGT poxvirus terminators, under the control of the respective VACV mH5, synthetic pLEO and modified fowlpox mFP promoters (35). This expression cassette was flanked by the 3’ ends of LSDV ORFs 49 and 50, and all genetic elements were amplified in the plasmid backbone pUC57-Simple (GenScript, China).

LSDV-SODis-p67HA-BLV-Gag was made by homologous recombination between the transfer vector pUC57-p67HA-BLV-Gag-mCherry and parent virus LSDV(SODis)BEFV-Gb (**Figure 1B**). LT cells were infected with LSDV(SODis)BEFV-Gb in DMEM at a range of multiplicities of infection (MOI) from 0.05 to 0.5, for two hours in 12-well plates. The media was removed and cells were subsequently transfected with 5 µg of pUC57-p67HA-BLV-Gag-mCherry linearized with KpnI and PacI (FastDigest, Thermo Fisher Scientific, USA), using 3 µl XtremeGENE™ HP (Roche, Switzerland) in DMEM. At two day’s post infection, cells were lysed by two freeze/thaw cycles (-80°C/37°C). The resulting lysates were passaged in MDBK cells and foci of MDBK cells that fluoresced red due to potential infection with LSDV-SODis-p67HA-BLV-Gag were physically scraped with a 10 µl pipette tip, placed into 100 µl DMEM in 1.5 ml microcentrifuge tubes, and lysed by two freeze/thaw cycles. Lysates were repeatedly passaged in MDBK cells until no green fluorescence (due to parent LSDV) was observed.

LSDV-SODis-p67HA-BLV-Gag was passaged twice in chick chorioallantoic membranes (CAMs) to remove any bovine viral diarrhea virus (BVDV) which may have been present in the MDBK cells. This was carried out as previously described (36). Further passages were performed in LT cells or BHK-21 cells to maintain BVDV-free stocks. Micrographs were obtained with AxioVert A.1 inverted fluorescence microscopes and Zen Blue 3.1 software (Zeiss, Germany).

### 2.3 Preparation of LSDV stocks

High titer stocks of LSDV-SODis-p67HA-BLV-Gag and nLSDVSODis-UCT were prepared by infecting MDBK, LT or BHK-21 cells in 175 cm^3^ flasks or HYPERFlasks^®^ (Corning^®^, USA) at MOIs 0.0025 to 0.005. Once all cells were infected and about 50% of cells had lifted, the flasks were frozen and thawed twice and lysates were clarified by low speed centrifugation at 320 x g for 10 min. Supernatants were placed into SS34 tubes and underlaid with 1-1.5 ml of 36% (w/v) sucrose diluted in 1X PBS. Viruses were pelleted by centrifugation at 27 000-39 000 x g for 1-2 hrs at 4°C. The pellets were resuspended in 1X PBS and stored at -80°C until needed. Prepared stocks were titrated by infecting MDBK cells in 96-well plates with serial dilutions of the stock (10^-1^ to 10^-12^ in DMEM) and tissue culture infectious dose at 50% infection (TCID_50_) was determined using the method described by Reed and Muench (37).

### 2.4 PCR confirmation of the insert

Amplification of the insert between ORFs 49 and 50 by polymerase chain reaction (PCR) was performed to confirm the presence of the expression cassette. DNA extracted from cells was used as template with forward (5’ GAGTGAAGCCTGGAACAT 3’) and reverse (5’ ACTCTATCGCATCTGGAAACT 3’) primers (17), Phusion^®^ High Fidelity DNA Polymerase (New England Biolabs, USA) and Phusion HF Buffer. The reaction parameters were as follows: initial denaturation at 98°C for 1 min, cycling conditions (25 cycles) of denaturation at 98°C for 30 s, annealing at 60°C for 30 s and extension at 72°C for 3 min, followed by a final single extension step at 72°C for 10 min. The products were resolved by electrophoresis in 0.8% agarose gels with 0.25 µg/ml ethidium bromide and 1X Tris borate EDTA (TBE) buffer.

### 2.5 Confirmation of p67HA and BLV Gag expression

The expression of p67HA and BLV Gag was confirmed by SDS PAGE and western blot analysis. MDBK cells or BHK-21 cells were infected with virus at MOIs 0.25-0.5. After 2-3 days, media was removed and cells were lysed with 200 µl Glo Lysis Buffer (Promega, USA) according to the manufacturer’s protocol. Media and lysates were clarified by centrifugation at 13 500 x g for 10 min, and the resulting supernatants were incubated at 95°C for 5 min in Laemmli buffer. Samples were separated in resolving gels containing 10% bis-acrylamide and detected as previously described (30). Goat anti-rabbit-IgG (A3687, Sigma, USA) and goat anti-mouse-IgG (ab97020, Abcam, UK), both conjugated to alkaline phosphatase, were used as secondary antibodies at 1:10 000.

### 2.6 Immunofluorescent staining of fixed and live cells

Immunostaining of fixed MDBK cells in 24-well plates infected with LSDV-SODis-p67HA-BLV-Gag at MOI 0.05 was performed as previously described at two days post-infection (30, 38). Live staining of HeLa cells infected with virus at MOI 0.05 in 4-well Permanox^®^ chamber slides (Thermo Fisher Scientific, USA) coated with poly-L-lysine (P8920, Sigma, USA) was performed at two days post infection as previously described (30, 38) using 1:100 anti-p67 antibody. Donkey anti-rabbit-IgG conjugated to Alexa Fluor 488 (Life Technologies, USA) was used as the secondary antibody at 1:1000.

### 2.7 Glycosylation status of p67ΔTM

N-linked and O-linked glycosylation sites in wild-type p67 (GenBank: AAA98601.1) - excluding the SS and anchor - were predicted with NetNGlyc-1.0 (39) and NetOGlyc-4.0 (40). Purified p67ΔTM was subjected to deglycosylation by treatment of 5 µg protein with PNGase F (New England Biolabs, USA) and 10 µg protein with Protein Deglycosylation Mix II (New England Biolabs, USA) according to the manufacturer’s protocol. SDS PAGE and western blots of the samples were carried out as described further above using 1:1000 anti-His antibody.

### 2.8 Electron microscopy

To isolate BLV Gag VLPs and poxvirions, MDBK cells in 75 cm^3^ flasks were infected with virus at MOIs 0.5 and 1. At three days post-infection, media from the flasks were harvested and fresh DMEM was added to each flask. Cells attached to the flasks were lysed in the fresh DMEM by two freeze/thaw cycles. The lysate and harvested media were clarified by centrifugation at 1260 x g for 10 min, placed into SS34 tubes, underlaid with 5 ml 12% OptiPrep™ (Sigma-Aldrich, USA) diluted in 1X Tris-buffered saline (TBS) and centrifuged at 48 000 x g for 1 hr at 4°C. Immunogold-labelling of VLPs and negative staining of pelleted poxvirus diluted 1:10 in TBS, with goat anti-rabbit-IgG conjugated to 10 nm colloidal gold (G7402, Sigma, USA) was performed as previously described (30). SDS PAGE and western blots were performed on isolated VLP samples as described earlier.

For ultra-thin sections, MDBK cells in 6-well plates were infected with virus at MOI 1. Two days post-infection, cells were fixed in the plate with 1 ml 2.5% glutaraldehyde diluted in PBS for 5 min at room temperature. Cells were scraped off the wells, placed into microcentrifuge tubes and centrifuged for 3 min at 15 900 x g. The pellets were resuspended in 1 ml PBS and centrifuged at 2350 x g for 2 min. The cell pellets were fixed a second time with 50 µl 2.5% glutaraldehyde for 1 hr at room temperature and washed twice with 100 µl PBS by pelleting at 2350 x g and resuspending. Low melting point agarose, 2% in H_2_O at 37°C, was added to cells and allowed to set. Samples were cut into 1 cm^3^ blocks, incubated in 0.5% tannic acid at room temperature for 1 hr, washed twice with PBS, fixed with 1% osmium tetroxide in PBS at room temperature for 1 hr, washed twice in PBS for 5 min and washed once in H_2_O for 5 min. Samples were dehydrated by an ethanol gradient: 30%, 50%, 70%, 80%, 90%, 95%, 100%, 10 min per percentage and incubated again in 100% ethanol for 10 min. The dehydrated samples were incubated twice in acetone for 10 min, overnight in 400 µl 1:1 acetone:resin (agar low viscosity resin, Agar Scientific, UK) and for 8 hrs in a 1:3 acetone: resin mixture. Samples were incubated overnight in 100% resin, replaced with fresh 100% resin, orientated into molds and set at 60°C for 24 hrs. Sections were placed on copper grids and stained with 2% uranyl acetate and lead citrate.

Grids were viewed by conventional transmission electron microscopy (TEM) with a Tecnai F20 microscope (Thermo Fisher (formerly FEI), Eindhoven, Netherlands) at the UCT Electron Microscope Unit.

### 2.9 Mouse immunizations

Mouse experiments were conducted at the UCT Research Animal Facility after approval by the UCT AEC for protocol AEC 020-020. Four groups of female BALB/c mice, five mice per group, were inoculated intramuscularly twice, 28 days apart, with 100 µl PBS (Thermo Fisher Scientific, USA), 100 µg pMExT-p67HA plasmid (30), 10^6^ ffu nLSDVSODis-UCT or 10^6^ ffu LSDV-SODis-p67HA-BLV-Gag. Plasmid and viruses were diluted in 100 µl PBS, and mice were injected with 50 µl inoculum per hind leg. End-bleeds were obtained by cardiac puncture on day 42 (PBS and pMExT-p67HA) and day 44 (nLSDVSODis-UCT and LSDV-SODis-p67HA-BLV-Gag).

### 2.10 Enzyme-linked immunosorbent assays

ELISAs to detect the presence of p67-binding and BLV Gag-binding antibodies in mouse sera were carried out as previously described using plates coated with purified p67ΔTM or purified BLV Gag (30). End-point titers were determined as the highest dilution that had an ELISA signal at least two-fold greater than that of the average PBS group 10^-1^ dilution reading. Data were analyzed in GraphPad Prism 5.0 (GraphPad, USA) whereby a one-way ANOVA and *post-hoc* Bonferroni test were conducted.

## 3 Results

### 3.1 Passage of LSDV-SODis-p67HA-BLV-Gag

**Figure 2:**
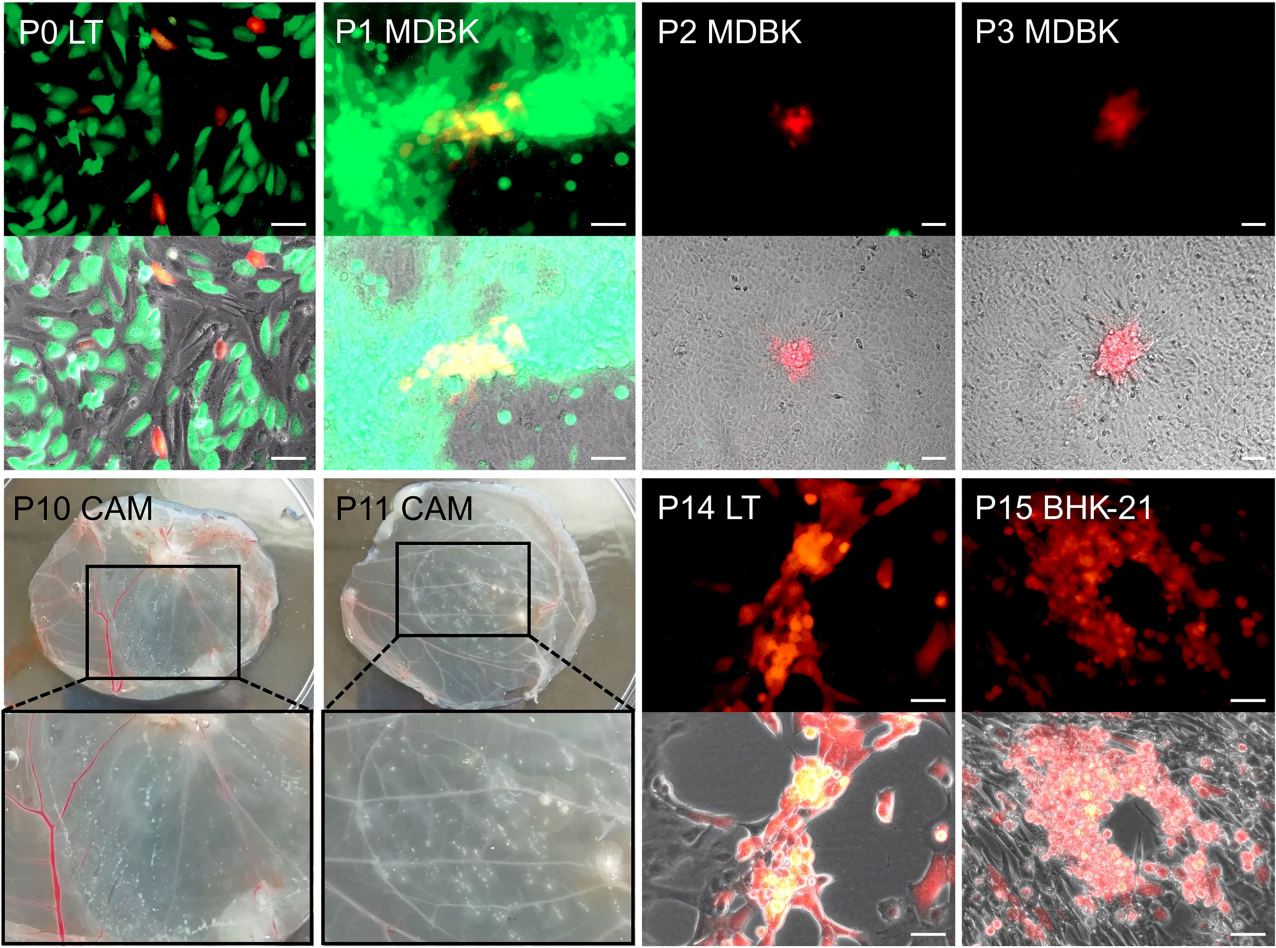
Generation and passage of LSDV-SODis-p67HA-BLV-Gag. The recombinant virus (mCherry; red) was constructed in LT cells at passage 0 (P0) by infection with LSDV(SODis)BEFV-Gb (eGFP; green) and transfection with the transfer vector. P1 MDBK = first passage of the lysate in MDBK cells, P2 MDBK = second passage in MDBK cells, P3 MDBK = third passage in MDBK cells. P10 CAM and P11 CAM show passage of the recombinant in CAMs of fertilized hens’ eggs. Magnified inserts show regions of LSDV white pocks. Later passages were performed in LT (P14 LT) and BHK-21 (P15 BHK-21) cells. Micrographs were taken using fluorescence only (upper panels) and fluorescence with phase (lower panels). All scale bars: 50 µm.

LSDV-SODis-p67HA-BLV-Gag was designed to express p67HA on the surface of BLV Gag VLPs to potentially enhance the immunogenicity of the p67 antigen (**Figure 1**). The fluorescent marker mCherry was included in the expression cassette to enable the recombinant virus to be distinguishable (red) from the parent virus (green) (**Figure 1B**). To construct the virus, at passage 0 (P0), LT cells were infected with the parent virus LSDV(SODis)BEFV-Gb and transfected with the transfer vector to enable homologous recombination to occur (**Figure 2**). The recombinant was isolated by physically picking red-fluorescing foci and passaging the resultant lysate in MDBK cells until no green fluorescence was seen - at P3. MDBK cells were used for this purpose as LSDV forms distinct foci, easy to pick, in this cell line. Unfortunately, MDBK cells harbour BVDV, a contaminating virus that cannot be present in LSDV stocks intended for cattle vaccination (36). Therefore, LSDV-SODis-p67HA-BLV-Gag was passaged twice in the CAMs of fertilized hens’ eggs at P10 and P11 to remove BVDV. Infected CAMs showed typical white pocks characteristic of LSDV infection. Further passages were performed in LT cells or BHK-21 cells to have stocks free from BVDV. BHK-21 cells were used for later passages, once this cell line was shown to be permissive for LSDV growth (35).

### 3.2 Verification and characterization of LSDV-SODis-p67HA-BLV-Gag

**Figure 3:**
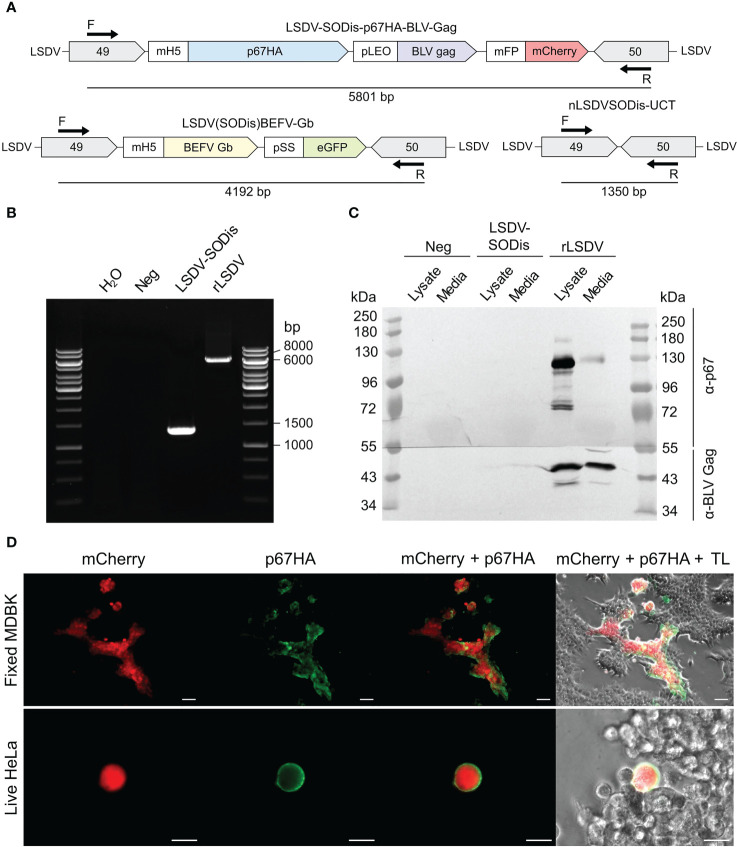
** (A)** Schematic diagrams of PCR product sizes expected to be amplified by the forward (F) and reverse (R) primers from LSDV-SODis-p67HA-BLV-Gag, the parent virus LSDV(SODis)BEFV-Gb, and the control virus nLSDVSODis-UCT which contained no insert. **(B)** Gel electrophoresis of PCR products from samples that had no template (H_2_O) or from DNA extracted from MDBK cells infected with no virus (Neg), nLSDVSODis-UCT (LSDV-SODis) or LSDV-SODis-p67HA-BLV-Gag (rLSDV). **(C)** SDS PAGE and western blot of samples from MDBK cells. The membrane was cut in half and probed with either anti-p67 antibody (α-p67) or anti-BLV-p24 (Gag) antibody (α-BLV Gag). **(D)** Immunofluorescent staining of fixed MDBK cells (scale bar: 50 µm) and live HeLa cells (scale bar: 20 µm), both infected with LSDV-SODis-p67HA-BLV-Gag at MOI 0.05, seen with mCherry, probed with anti-p67 antibody and a secondary antibody conjugated to Alexa Fluor 488 (green). TL: Transmitted light.

The insert between LSDV ORFs 49 and 50 was amplified by PCR to confirm the presence of the expression cassette (**Figures 3A, B**). LSDV-SODis-p67HA-BLV-Gag gave a product slightly larger than the expected 5801 bp size (**Figure 3B**), however sequencing showed that this product was correct. The expected product was seen for the nLSDVSODis-UCT positive control, and no LSDV(SODis)BEFV-Gb parent virus was detected.

SDS PAGE and western blotting confirmed the expression of the p67HA and BLV Gag proteins from cells infected with LSDV-SODis-p67HA-BLV-Gag (**Figure 3C**). BLV Gag was detected in lysate and media near the expected 43 kDa size, whereas p67HA which translates to 77 kDa was observed as multiple proteins with a predominant form slightly below 130 kDa in the lysate. Immunofluorescent staining of fixed infected MDBK cells further confirmed the expression of p67HA as seen by the overlap of green and red fluorescence (**Figure 3D**). Live cell immunofluorescent staining was performed, as incorporation into BLV Gag VLPs requires the antigen to be present at the plasma membrane. Surface localization of p67HA was confirmed on infected HeLa cells (**Figure 3D**).

### 3.3 Electron microscopy of LSDV-SODis-p67HA-BLV-Gag virions and BLV Gag VLPs

**Figure 4:**
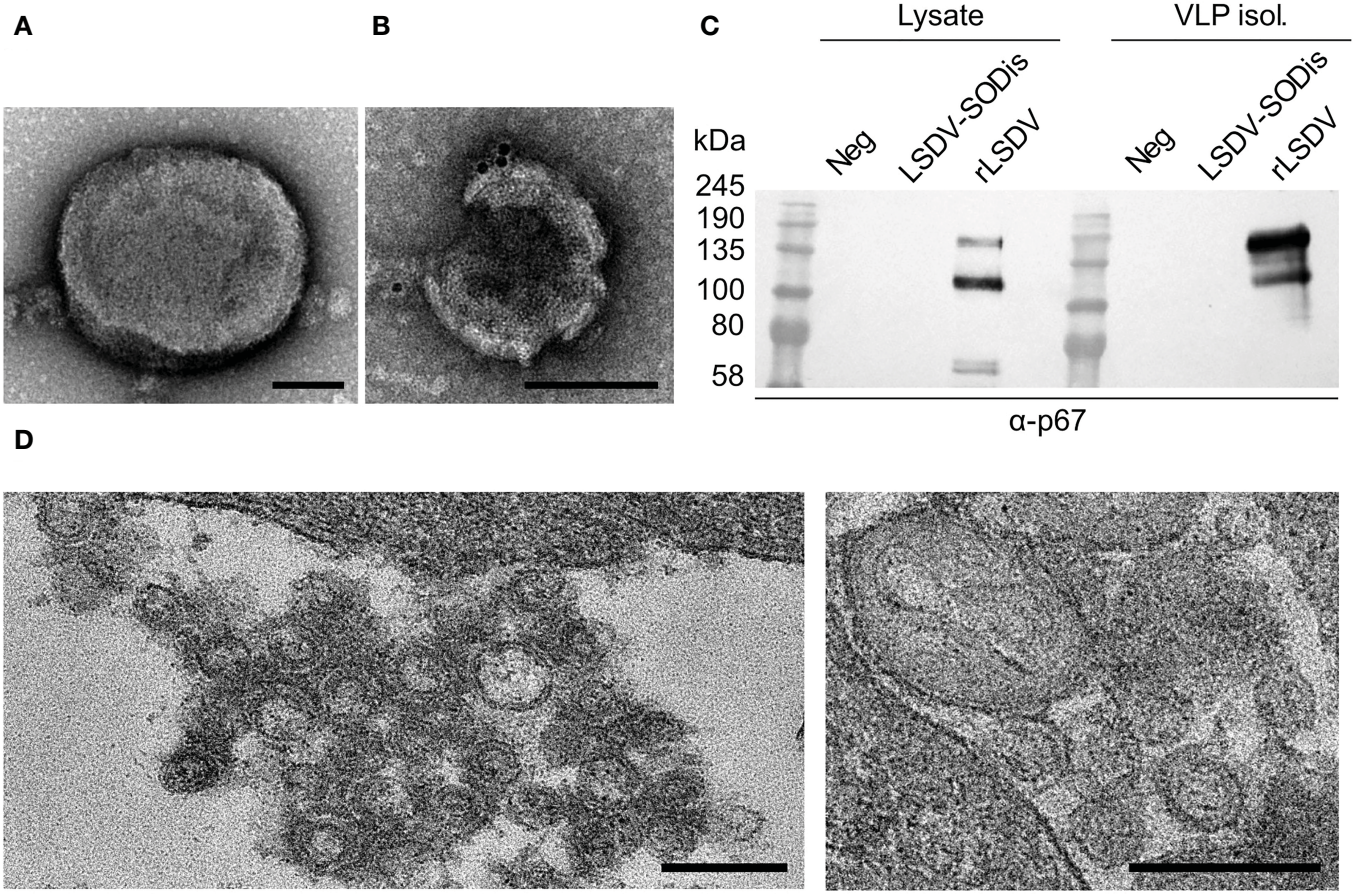
TEM of **(A)** LSDV-SODis-p67HA-BLV-Gag pox virion isolated from MDBK cells; **(B)** BLV Gag VLP isolated from infected MDBK cell media with immunogold labelling of p67HA (10 nm colloidal gold); both scale bars: 100 nm. **(C)** SDS PAGE and western blot of samples corresponding to **(A, B)** either uninfected MDBK cells (Neg), or infected with nLSDVSODis-UCT (LSDV-SODis) or LSDV-SODis-p67HA-BLV-Gag (rLSDV). VLP isol.: VLP isolation samples from cell media. **(D)** Potential BLV Gag VLPs in thin sections of MDBK cells infected with LSDV-SODis-p67HA-BLV-Gag. Scale bars: 250 nm.

Conventional TEM was performed to confirm BLV Gag VLP formation when expressed from cells infected with LSDV-SODis-p67HA-BLV-Gag and to investigate pox virion morphology. Isolated pox virions had characteristics typical of intracellular LSDV (**Figure 4A**). BLV Gag VLPs purified from the corresponding cell media samples appeared as expected (**Figure 4B**), however low numbers of VLPs were isolated. Low numbers of gold particles to detect p67HA on VLPs were also observed. SDS PAGE and western blotting of the cell lysate and isolated VLP samples showed that p67HA was detected in both samples (**Figure 4C**). In the VLP sample, the majority of p67HA appeared as a higher molecular weight protein (>135 kDa), whereas the predominant form in the lysate was closer to 100 kDa. Ultra-thin sectioning was performed on infected MDBK cells to further confirm BLV Gag VLP formation (**Figure 4D**). Areas near the plasma membrane and between adjacent cells contained circular structures which resembled VLPs. These were not seen in sectioned MDBKs infected with nLSDVSODis-UCT or uninfected MDBK cells (not shown). LSDV-SODis-p67HA-BLV-Gag observed in sections showed typical poxvirus morphologies.

### 3.4 Glycosylation status of p67ΔTM

**Figure 5:**
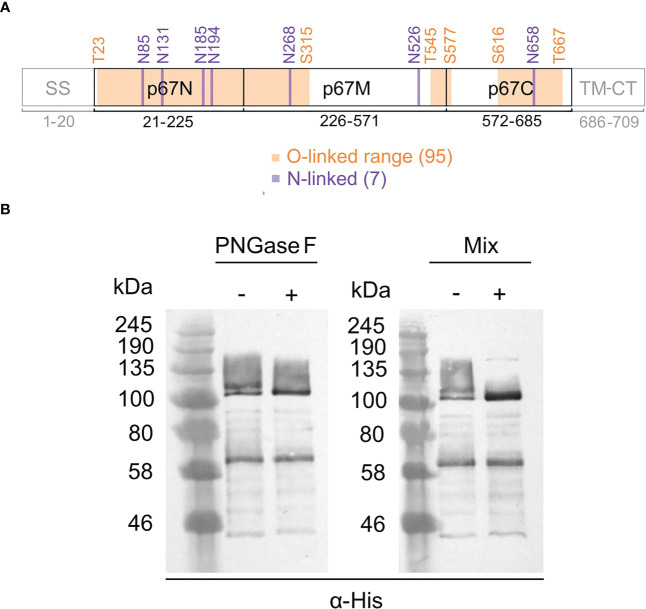
Glycosylation of p67ΔTM. **(A)** Predicted N-linked and O-linked glycosylation sites on p67 regions identical to wild-type p67 (GenBank: AAA98601.1), p67HA and p67ΔTM. O-linked sites are depicted as ranges. Labelled amino acid residue positions correspond to those in wild-type p67. **(B)** SDS PAGE and western blot of purified p67ΔTM protein treated (+) with PNGase F to remove N-linked glycans or a protein deglycosylation mix (Mix) to remove both N-linked and O-linked glycans or left untreated (-). Lanes were loaded with 40 ng of protein and blots were probed with anti-His antibody (α-His).

The presence of glycans on a protein can affect its mobility during SDS PAGE and give rise to additional bands on a western blot. As this may explain the different sizes of p67 protein seen on blots, such as in **Figure 4C**, we investigated the presence of N-linked and O-linked glycans on the protein. The soluble p67ΔTM protein (**Figure 1A**) was used for this purpose as it was purified from cell media and provided a relatively clean sample (30). The p67 regions that are identical amongst wild-type p67, p67HA and p67ΔTM were analyzed with NetNGlyc-1.0 and NetOGlyc-4.0 to predict the glycosylation sites (**Figure 5A**). Seven N-linked sites were predicted, as was previously described by others (41, 42), and 95 O-linked sites were predicted, shown as a range (exact sites shown in **Table S1**).

p67ΔTM was deglycosylated with PNGase F to remove N-linked glycans and treated with a deglycosylation mix to remove both N-linked and O-linked glycans (**Figure 5B**). The doublet >100 kDa resolved into one band after treatment with PNGase F, indicating that p67ΔTM has N-linked glycans. The band and smear up to ~160 kDa all resolved into a single band following treatment with the deglycosylation mix, indicating that the higher molecular weight protein is heavily O-linked glycosylated. The lower molecular weight form above 58 kDa had no mobility shift.

### 3.5 Immunogenicity of LSDV-SODis-p67HA-BLV-Gag in mice

**Figure 6:**
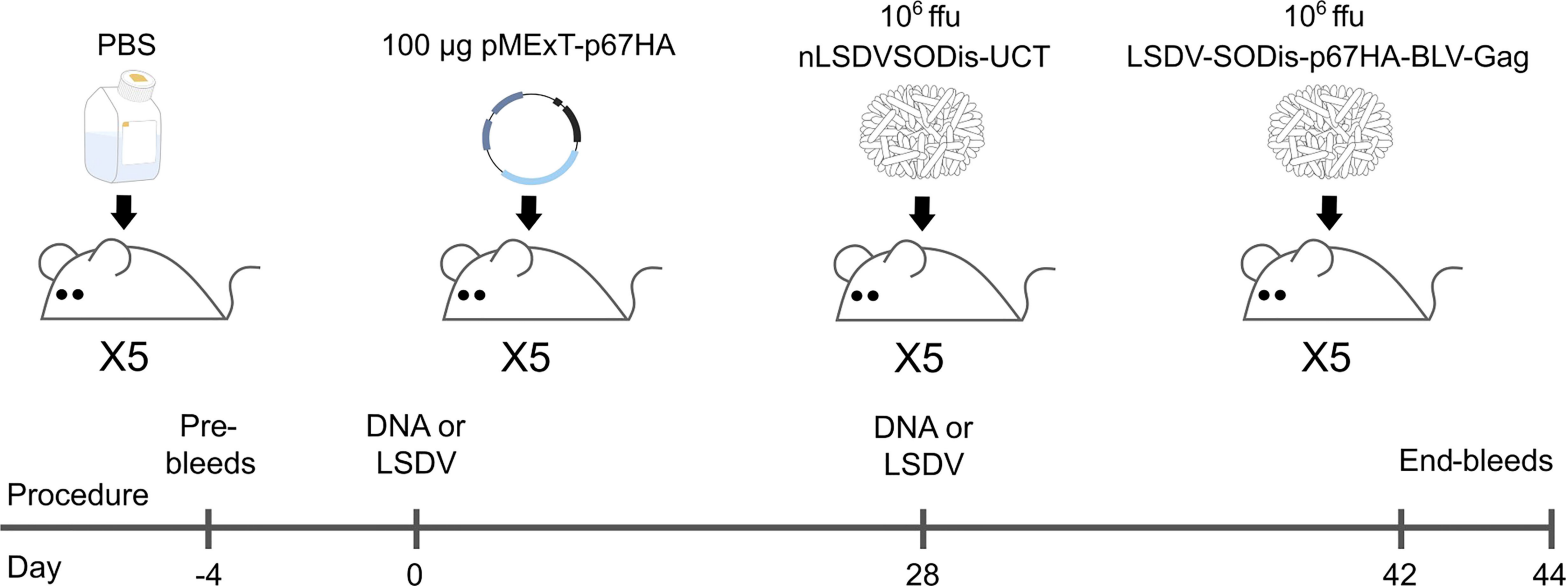
Inoculation and bleed schedule for BALB/c mice injected intramuscularly. End-bleeds were performed for the PBS and DNA groups on Day 42, and for the LSDV groups on Day 44.

The ability of LSDV-SODis-p67HA-BLV-Gag to elicit humoral responses against p67HA and BLV Gag was investigated in mice. Four groups of female BALB/c mice were inoculated as shown in **Figure 6**. The DNA plasmid pMExT-p67HA was used as a control, as we had previously shown this plasmid to induce p67-binding antibodies in mice after four inoculations (30). The plasmid consists of the pTHpCapR/pMExT mammalian expression vector backbone (38, 43) encoding the p67HA antigen present in LSDV-SODis-p67HA-BLV-Gag (**Figure 1A**).

**Figure 7:**
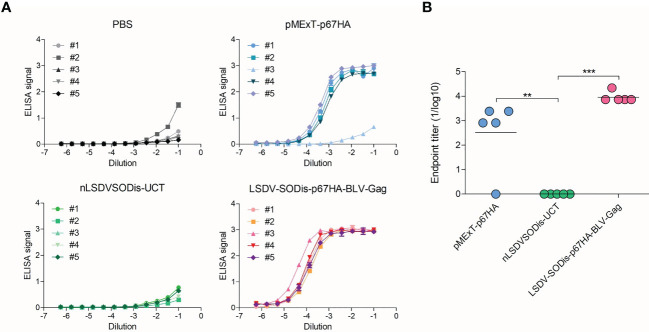
ELISAs to detect p67-binding IgG antibodies in vaccinated mouse sera. **(A)** ELISA signal (absorbance at 450-540 nm) vs Dilution for each mouse in each group, where a number refers to individual mice. Readings are shown with ± SEM. **(B)** End-point titers (1/log10) for each group. Values were set to zero if no response was observed. Values for each mouse are shown with the mean, with **p<0.01 and ***p<0.001 after a one-way ANOVA and Bonferroni *post-hoc* test.

An ELISA using plates coated with p67ΔTM showed that both pMExT-p67HA and LSDV-SODis-p67HA-BLV-Gag elicited p67-binding antibodies in mice (**Figure 7A**). Mouse #3 in the pMExT-p67HA group had no response, and why this occurred is unknown. Mice inoculated with LSDV-SODis-p67HA-BLV-Gag had endpoint titers almost 10-fold higher compared to those inoculated with pMExT-p67HA, however the differences were not statistically significant (**Figure 7B**).

**Figure 8:**
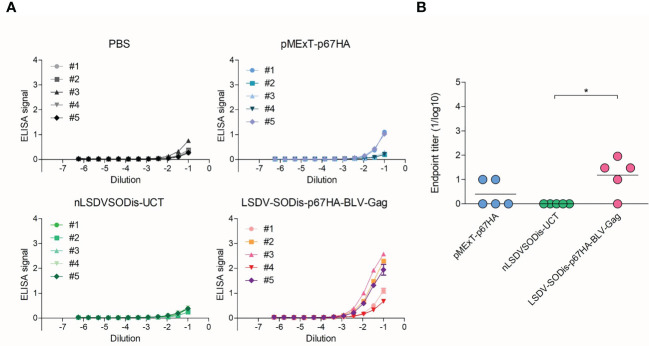
ELISAs to detect BLV Gag-binding IgG antibodies in vaccinated mouse sera. **(A)** ELISA signal (absorbance at 450-540 nm) vs Dilution for each mouse in each group, where a number refers to individual mice. Readings are shown with ± SEM. **(B)** End-point titers (1/log10) for each group. Values were set to zero if no response was observed. Values for each mouse are shown with the mean, with *p<0.05 after a one-way ANOVA and Bonferroni *post-hoc* test.

Responses to BLV Gag were investigated using plates coated with BLV Gag protein purified from *E. coli* (30). LSDV-SODis-p67HA-BLV-Gag elicited low titers of BLV Gag-binding antibodies, which were significantly different from the nLSDVSODis-UCT control group but not to those that received pMExT-p67HA (**Figure 8**). A summary of the endpoint titers can be found in **Table S2**.

## 4 Discussion

The vaccine currently in use against ECF is effective at protecting cattle, however, has many limitations that can be improved upon. Our goal was to produce a novel vaccine that may address these issues by combining our p67HA-VLP system with our recombinant nLSDVSODis-UCT backbone to develop a dual vaccine that potentially protects against both ECF and LSD (26, 30).

Most efforts to produce a novel vaccine that avoids the use of live *T. parva* have investigated the use of the p67 antigen, as it is conserved across many *T. parva* strains and can induce neutralizing antibodies to prevent sporozoite invasion (33). These have included chimeric p67 recombinants, truncation of the protein to known immunogenic regions, and expression in bacteria, insect cells *via* recombinant baculovirus, and mammalian cell expression systems (28, 42, 44). A peculiar characteristic of the antigen is its mobility after SDS PAGE and western blotting. The predominant protein extracted from sporozoite lysate appears to be 67 kDa, however recombinant forms as described earlier all tend to produce p67 at a range of sizes, often showing much larger proteins predominating in cell media (33, 42, 45, 46). This characteristic was observed for p67HA when expressed from LSDV (**Figures 3C**; **4C**). We have previously seen this pattern of expression for p67HA, p67ΔTM and p67 with the native TM-CT when expressed in HEK293T cells using the pMExT DNA plasmid (30). Tebaldi et al. (42) have demonstrated that larger soluble ~140 kDa forms are possibly p67 aggregates that only dissociate under severe denaturation conditions. p67 has one cysteine residue (C416) in p67M, therefore the formation of dimers *via* disulphide bonds is not impossible. p67HA also showed similar large proteins in blots; one slightly above 100 kDa and another at ~140 kDa (**Figure 4C**). As p67ΔTM showed a very similar pattern of expression, the protein purified from HEK293T cell media was used to investigate p67 glycosylation. Deglycosylation experiments showed that the antigen has both N-linked and O-linked glycans (**Figure 5B**). Treatment with PNGase F gave a result that was almost identical to that of Nene et al. (45); Kaba et al. (46), where they treated insect cells expressing p67 through baculovirus, with or without the TM respectively, with tunicamycin to prevent N-linked glycosylation of the protein. Interestingly, Tebaldi et al. (42) had a conflicting result where treatment of their p67ΔTM from HEK293T cell media with PNGase F showed no mobility shift. They, however, used the native SS whereas we used the TPA SS, and different SS can result in differences in glycosylation (47, 48). If one compares p67HA in the lysate versus the medium (VLPs), the opposite band predominates for the different samples (**Figure 4C**). If the glycosylation status of p67ΔTM is taken into account, it is likely that p67HA present on the cell surface and VLPs is the most processed, glycosylated form, whereas p67HA in the lysate represents the protein before undergoing processing. Processing and the addition of glycans progresses as proteins are trafficked through the endoplasmic reticulum and Golgi body, therefore one would expect the most processed form to be on the cell surface (49). Besides explaining the appearance of p67 on western blots, glycosylation may have conformational and immunological implications. For some viruses, such as HIV, neutralizing antibodies have epitopes that are dependent on the presence of glycans. Conversely, glycan shielding of epitopes can also occur whereby the presence of glycans sterically blocks the binding of antibodies, as seen for the heavily glycosylated Ebola virus glycoprotein (50). Whether these factors are relevant for p67 is currently unknown. As yet, no glycans have been found experimentally on sporozoite-derived p67 (41). Thus, one can hypothesize that non-glycosylated p67 maybe the most suitable form for eliciting neutralizing antibodies to *T. parva* sporozoites.

The expression of p67HA on the surface of HeLa cells was shown by immunofluorescence, and electron microscopy confirmed the presence of BLV Gag VLPs (**Figures 3D**; **4**). However, the amount of p67HA on the surface of VLPs was considerably lower compared to when expressed from DNA plasmids, which employed the constitutive cytomegalovirus (CMV) promoter (30). The mH5 and pLEO promoters, which were used for the control of p67HA and BLV Gag respectively, both induce gene expression at the early and late stages of the poxvirus life cycle (51, 52). However, different promoters can have different strengths at different times, and therefore the timing of p67HA and BLV Gag expression may be slightly different (53). Furthermore, the production of p67HA-BLV Gag VLPs would require both proteins to be present at the same assembly point, which may be affected by differential trafficking of the two proteins through the cytoplasm of recombinant LSDV-infected cells. Isolation of VLPs was also met with difficulties as low numbers of VLPs were observed. VLP isolation was also met with technical challenges with a different recombinant nLSDVSODis-UCT encoding HIV-1 Gag under the pLEO promoter (unpublished data). HIV-1 Gag only associates with the plasma membrane after a threshold cytoplasmic concentration of the protein is reached (54). This may explain the lower numbers of isolated VLPs from cell media if relatively lower expression of Gag from LSDV occurred.

Nevertheless, LSDV-SODis-p67HA-BLV-Gag elicited p67-binding antibodies in mice with an average titer that was almost 10-fold higher than that of the pMExT-p67HA DNA plasmid, although the difference was not statistically significant (**Figure 7**). Live-virus vaccines often induce stronger responses than DNA vaccines used alone, therefore this result was expected. Others have demonstrated recombinant p67 immunogenicity in mice, which then proved immunogenic in cattle. Kaba et al. (29) showed that when inoculated into mice, p67ΔSS fused to GFP (GFP: p67ΔSS, produced in insect cells), and p67C fused to baculovirus GP64 and displayed on baculovirus particles (GP64:p67C) resulted in antibody titers over 5-fold higher than that of other truncated p67 proteins (GFP:p67C/p67N, p67C produced in *E. coli*, and GP64:p67N). These two antigens also induced *T. parva* sporozoite neutralizing antibodies in mice, and GFP:p67ΔSS was the superior antigen in their cattle immunogenicity study.

Surprisingly, mice inoculated with LSDV-SODis-p67HA-BLV-Gag had relatively low responses to BLV Gag (**Figure 8**). This was unexpected as the DNA plasmid pMEx-BLV-gag, which encoded the same gag gene, previously gave good responses in mice (30). The implication is that the immune response to p67 was not linked to that of BLV Gag and that the increased p67HA antibody response may have been due to the p67 being presented on poxvirus particles and not predominantly on BLV VLPs. The lower level of detection of p67HA on LSDV-produced Gag particles would support this theory. As described earlier, the pLEO promoter is a synthetic early-late optimized promoter (52) which should be expressed before and after LSDV DNA replication. The expression was reported to be high in the first hour resulting in good CD8+ T-cell responses, so another possibility is that a cellular immune response is being activated.

In conclusion we have constructed a novel LSDV candidate ECF vaccine expressing a chimeric protein p67HA expressed on the surface of infected cells and BLV Gag. We further show that our p67 is glycosylated when expressed in mammalian cells, which may explain the multiple large proteins observed in western blot analysis. LSDV-SODis-p67HA-BLV-Gag elicited p67 and BLV-Gag binding antibodies in mice. This vaccine is currently being investigated in cattle to assess its efficacy against ECF.

## Data availability statement

The original contributions presented in the study are included in the article/**Supplementary Material**. Further inquiries can be directed to the corresponding author.

## Ethics statement

The animal study was reviewed and approved by the Animal Ethics Committee of the University of Cape Town.

## Author contributions

LW: methodology, investigation, data curation, formal analysis and writing (original draft, review and editing). RC: methodology, supervision and writing (review and editing). ND: methodology, investigation and writing (review and editing). MJ: methodology and investigation. EM: methodology and investigation. ER: conceptualization, supervision and writing (review and editing). A-LW: conceptualization, supervision, methodology, funding acquisition and writing (review and editing). All authors contributed to the article and approved the submitted version.
