# Supplementary material for: Development of a dual vaccine against East Coast fever and lumpy skin disease
Source: Front Immunol. 2023 Mar 30;14:1143034. doi: 10.3389/fimmu.2023.1143034 (PMC10098110; doi:10.3389/fimmu.2023.1143034)
Supplement: Supplementary file 1 [file Table_1.docx]

**Table S1.** O-linked glycosylation sites in regions identical to wild-type p67 (GenBank: AAA98601.1), p67HA and p67ΔTM predicted by NetOGlyc-1.0. The amino acid position and score out of 1 for each site are shown. Positions are relative to wild-type p67.

| **Position** | **Score** | **Position** | **Score** | **Position** | **Score** | **Position** | **Score** |
| --- | --- | --- | --- | --- | --- | --- | --- |
| 23 | 0.72 | 105 | 0.84 | 197 | 0.96 | 315 | 0.67 |
| 30 | 0.96 | 107 | 0.85 | 198 | 0.95 | 545 | 0.60 |
| 37 | 0.96 | 109 | 0.95 | 199 | 0.95 | 547 | 0.55 |
| 40 | 0.96 | 114 | 0.95 | 208 | 0.98 | 556 | 0.83 |
| 43 | 0.99 | 116 | 0.95 | 214 | 0.96 | 559 | 0.96 |
| 46 | 0.99 | 128 | 0.94 | 215 | 0.99 | 564 | 0.91 |
| 50 | 0.96 | 133 | 0.91 | 216 | 0.99 | 565 | 0.92 |
| 53 | 0.95 | 135 | 0.88 | 219 | 0.99 | 567 | 0.93 |
| 66 | 0.93 | 142 | 0.93 | 220 | 0.95 | 573 | 0.82 |
| 69 | 0.99 | 143 | 0.95 | 221 | 0.98 | 577 | 0.74 |
| 70 | 0.94 | 145 | 0.96 | 227 | 0.88 | 616 | 0.55 |
| 71 | 0.98 | 146 | 0.96 | 232 | 0.94 | 617 | 0.68 |
| 74 | 0.99 | 149 | 0.99 | 235 | 0.97 | 622 | 0.84 |
| 75 | 0.98 | 151 | 0.99 | 239 | 0.90 | 626 | 0.74 |
| 76 | 0.98 | 154 | 0.98 | 241 | 0.90 | 630 | 0.71 |
| 79 | 0.98 | 157 | 0.99 | 243 | 0.96 | 637 | 0.90 |
| 82 | 0.98 | 162 | 0.97 | 244 | 0.84 | 640 | 0.86 |
| 87 | 0.92 | 164 | 0.98 | 258 | 0.94 | 642 | 0.91 |
| 88 | 0.97 | 169 | 0.95 | 264 | 0.98 | 645 | 0.94 |
| 89 | 0.97 | 179 | 0.95 | 267 | 0.95 | 655 | 0.98 |
| 92 | 0.87 | 184 | 0.94 | 269 | 0.91 | 660 | 0.94 |
| 94 | 0.97 | 187 | 0.87 | 270 | 0.96 | 662 | 0.93 |
| 101 | 0.97 | 189 | 0.60 | 299 | 0.98 | 667 | 0.86 |
| 103 | 0.93 | 196 | 0.94 | 304 | 0.96 |  |  |

**Table S2.** Detection of p67- and BLV Gag-binding antibodies in mouse sera by ELISA. Endpoint titers of each mouse was determined by taking the next reciprocal dilution 2-fold higher than the mean of the PBS group at 10^-1^. The endpoint titre was set to zero if no dilution gave values higher than the cut off. Negative responses are shaded grey.

| **Vaccine** | **Mouse** | **Endpoint titer** | | **Vaccine** | **Mouse** | **Endpoint titer** | |
| --- | --- | --- | --- | --- | --- | --- | --- |
|  |  | **p67** | **BLV Gag** |  |  | **p67** | **BLV Gag** |
| pMExT-p67HA | #1 | 2430 | 10 | nLSDVSODis-UCT | #1 | 0 | 0 |
|  | #2 | 810 | 0 |  | #2 | 0 | 0 |
|  | #3 | 0 | 0 |  | #3 | 0 | 0 |
|  | #4 | 810 | 0 |  | #4 | 0 | 0 |
|  | #5 | 2430 | 10 |  | #5 | 0 | 0 |
| LSDV-SODis-p67HA-BLV-Gag | #1 | 7290 | 10 |  |  |  |  |
|  | #2 | 7290 | 30 |  |  |  |  |
|  | #3 | 21870 | 90 |  |  |  |  |
|  | #4 | 7290 | 0 |  |  |  |  |
|  | #5 | 7290 | 30 |  |  |  |  |
